# Analysis and Comparison of Early Childhood Nutritional Outcomes Among Offspring of Chinese Women Under the Chinese 2021 and US 2009 Gestational Weight Gain Guidelines

**DOI:** 10.1001/jamanetworkopen.2022.33250

**Published:** 2022-09-23

**Authors:** Fangfang Chen, Peng Wang, Jing Wang, Zijun Liao, Xinnan Zong, Yiren Chen, Jianqiang Lai, Ting Zhang, Gongshu Liu, Xianghui Xie

**Affiliations:** 1 Capital Institute of Pediatrics, Beijing, China; 2 Tianjin Women’s and Children’s Health Center, Tianjin, China; 3 National Institute of Nutrition and Health, Chinese Centre for Disease Control and Prevention, Beijing, China

## Abstract

**Importance:**

In 2009, the US National Academy of Medicine (NAM) released revised gestational weight gain (GWG) guidelines, which were established primarily for White North American women and may be unsuitable for Asian women. In 2021, the Chinese Nutrition Society (CNS) released its GWG guidelines, but their applicability requires re-examination.

**Objective:**

To compare the differences between the CNS and NAM recommendations for GWG in association with health outcomes in the offspring of Chinese women.

**Design, Setting, and Participants:**

In this bidirectional cohort study, children in China were recruited at age 3 years from 2017 to 2018, with 2 follow-up visits over the next 2 years (between September 2017 and September 2020). Information during pregnancy was retrieved from medical records. Data analysis was performed from October 2021 to January 2022.

**Main Outcomes and Measures:**

GWG was classified as insufficient, appropriate, or excessive according to the CNS and NAM guidelines separately. Children’s height, weight, fat mass, fat-free mass, and percentage of body fat were measured at each visit. Body mass index, fat mass index, fat-free mass index, weighted κ score, risk ratio values, sensitivity, specificity, positive predictive value (PPV), and negative predictive value (NPV) were calculated.

**Results:**

A total of 3822 children (1996 boys and 1826 girls; mean [SD] age, 3.79 [0.30] years) were enrolled; after exclusions, 3170 term singleton children were recruited and were followed at 4 and 5 years of age. According to the CNS guidelines, the prevalence rates were 14.1% for insufficient GWG, 48.1% for appropriate GWG, and 37.9% for excessive GWG, whereas the rates according to NAM guidelines were 39.7% for insufficient GWG, 37.2% for appropriate GWG, and 23.1% for excessive GWG. The weighted κ value for the classification agreement between the 2 guidelines was 0.530 (95% CI, 0.510-0.550). For the appropriate GWG group, the rates for low nutritional levels did not differ between the 2 guidelines, but the rates for high nutritional levels were significantly lower under CNS guidelines than under NAM guidelines. When the sensitivity, specificity, PPV, and NPV with respect to the mothers who maintained appropriate GWG were used to estimate the nonhigh nutritional status of their offspring, generally higher values based on the CNS guidelines were found compared with those based on the NAM recommendations.

**Conclusions and Relevance:**

These findings suggest that the GWG recommendations promulgated by the NAM are higher than the CNS guidelines, with the latter more suitable for Chinese women.

## Key Points

**Question:**

Which gestational weight gain (GWG) guideline for Chinese women is associated with preferred nutritional outcomes among their offspring: Chinese Nutrition Society (CNS) 2021 or US National Academy of Medicine (NAM) 2009 guidelines?

**Findings:**

In this cohort study, compared with the NAM guidelines, GWG concordant with the CNS 2021 guidelines was associated with a lower prevalence of macrosomia, obesity, and high fat mass at ages 3 to 5 years in this cohort of Chinese women and offspring. There was no increase in undernutrition.

**Meaning:**

These findings suggest that the GWG recommendations of CNS 2021 may be more suitable for Chinese women than the NAM guidelines.

## Teaser

This cohort study examines the differences between the 2021 Chinese Nutrition Society and 2009 US National Academy of Medicine recommendations for gestational weight gain in association with health outcomes in the offspring of Chinese women.

## Introduction

Inappropriate gestational weight gain (GWG) may be associated with postpartum depression,^1,2^ cardiometabolic risk,^3^ and weight retention by the mother,^4,5,6^ in addition to presenting untoward consequences with regard to birth outcomes,^7,8^ including preeclampsia, gestational hypertension, gestational diabetes, cesarean delivery, preterm birth, small or large size with respect to gestational age at birth,^9^ and unfavorable long-term child health.^10,11,12,13,14^ In 2009, the US National Academy of Medicine (NAM, formerly the Institute of Medicine) released revised GWG guidelines^15^ that have been widely adopted in many countries. Previous studies have shown that adherence to the NAM recommendations appears to achieve improved pregnancy outcomes^16^ and that GWG outside the NAM recommendations may be associated with adverse pregnancy outcomes and unhealthy children’s status.^17,18,19,20,21^

It should be noted that obesity is excess adipose tissue and not excess weight.^22^ Asian individuals possess a higher percentage of body fat at a lower body mass index (BMI; calculated as weight in kilograms divided by height in meters squared), and the ethnic specificity in fat store patterns is partially determined by genetics.^23^ The association of adiposity with diabetes risk differs by race, as demonstrated by genetic studies,^24^ and the associations among race, ethnicity, and GWG are unduly complex.^25^ There is a wide variety of guidelines for GWG in different countries worldwide, with the majority of these countries proposing GWG ranges that are the same as or similar to the NAM guidelines.^26^ However, the NAM guidelines retain some limitations,^27,28,29^ including too large a range of recommendations for women with obesity.^30,31^ Moreover, the establishment of NAM guidelines was principally based on US White women, which limits their generalizability to other racial populations^32^ and makes them unsuitable for Latino^33^ and Asian populations.^34^ Additionally, we recognize that there is a paucity of publications regarding the utility of NAM guidelines among Asian women.^35^

In October 2021, the Chinese Nutrition Society (CNS) released the group standard Weight Monitoring and Evaluation During Pregnancy Period of Chinese Women (shown in English in eTable 1 in the Supplement)^36^ based on data from a cohort that included more than 100 000 pregnant Chinese women. Considering the association of GWG with short-term and long-term child health outcomes that include preschool child adiposity,^37^ and given the present study’s bidirectional cohort of offspring birth outcomes and body composition follow-up visits for children aged 3 to 5 years, here we compared the differences in GWG recommendations between CNS 2021 and NAM 2009 in screening and early warning aspects regarding nutritional levels in the offspring of Chinese women. We hope our results can clarify whether the GWG recommendations provided by the CNS are more suitable for Chinese women than the NAM guidelines.

## Methods

This cohort study (the Kindergarten Cohort) was implemented by the Tianjin Women’s and Children’s Health Center and Capital Institute of Pediatrics in Tianjin from 2017 to 2020, and it was approved by the institutional review board of Tianjin Women’s and Children’s Health Center.^37^ This report followed the Strengthening the Reporting of Observational Studies in Epidemiology (STROBE) reporting guidelines for cohort studies.

### Participants

In China, children generally enter kindergarten at age 3 years and stay there for 3 years (ie, junior, middle, and senior classes) before elementary school. By using a stratified cluster-sampling method, we selected 11 districts (including 6 central urban districts, 4 loop urban districts, and 1 suburban district) from the 16 municipal districts in Tianjin, and 42 kindergartens were selected from the 11 districts beginning in 2017. Children in the first year of the 42 kindergartens (3-year-olds) were recruited to the cohort study, and they were followed up and completed their physical examinations in their second and third years between September 2017 and September 2020. The original objectives of the Kindergarten Cohort were to describe the growth of children and the status of obesity-related metabolic disorders, and its study size was decided according to the prevalence of obesity.

Parental written consent forms were obtained when the children were recruited to the junior class. Children’s birth weights and their mothers’ clinical information regarding antenatal and postnatal health care were retrieved from records in the Tianjin Hospital Healthcare Medical System and included anthropometric results collected at each follow-up phase. The exclusion criteria regarding the children were as follows: (1) an inability to obtain informed consent from their parents, (2) presenting with any condition or chronic disease or use of any drug known to affect growth and development, (3) the presence of acute diseases that prohibit children from participating in the physical examination (4) twins or other multiple births, and (5) mothers without GWG records in the antenatal health care system.

### Measurements of Gestational Weight

Prepregnancy weight and height were self-reported and registered at the first visit during the first trimester, and maternal gestational weight was measured at antenatal clinics during each visit. The median (IQR) number of repeated weight measurements taken per woman was 7 (6-8). GWG was calculated as weight at the last visit minus the self-reported weight for the first visit at the antenatal clinic.

### Anthropometric Measurements of Children

Height was measured without shoes at each visit for each child followed up in kindergarten. Body weight, fat mass, fat-free mass, and percentage of body fat mass (FM%) were measured by trained nutritionists using bioelectrical impedance analysis (BIA) with a Seehigher H-Key 350 device (Beijing Seehigher Technology Co, Ltd). Fat mass index (FMI) and fat-free mass index (FFMI) were also calculated for each subject as fat mass and fat-free mass in kilograms divided by height in meters squared, respectively. To address potential sources of bias in our analysis, we collected and evaluated information on prepregnancy BMI, maternal age at delivery, the mother’s and father’s careers, mother’s and father’s educational levels, annual family income, parity, gestational age, and whether the mother had breast-fed for 6 months.

### Classification of Health Outcomes and Conditions

GWG was classified as insufficient, appropriate, or excessive according to the respective CNS 2021 and NAM 2009 guidelines.^15^ Full-term infants were defined as those born at gestational age greater than or equal to 37 weeks and less than 42 weeks,^38^ low birth weight was defined as birth weight less than 2500 g, and macrosomia was defined as birth weight greater than or equal to 4000 g. Obesity was diagnosed according to the World Health Organization (WHO) reference value,^39,40,41^ and *z* scores were also evaluated according to the WHO reference, including the *z* scores for height-for-age, weight-for-height, and BMI-for-age.

The areas under the curve (AUCs) for the 3-year body composition measurements during follow-up were calculated to indicate the overall adiposity level in kindergarteners. To compute the AUC for each individual, we established quadratic growth curves of the 3 measurements of adiposity indicators in kindergarten children in each group with respect to sex and age by using a random-effects model with SAS PROC MIXED.^42^ Low AUC-FFMI was defined as an age-specific and sex-specific FFMI *z* score less than −1; high AUC-FM% and high AUC-FMI were defined as *z* scores 1 or greater; low nutritional levels of the children were defined as a *z* score less than −1, with low AUC-FFMI and low birth weight; and high nutritional levels of the children included macrosomia, a *z* score 1 or higher, and high AUC-FM% and high AUC-FMI.

### Statistical Analysis

Data analysis was performed from October 2021 to January 2022. Data analyses were conducted using SPSS statistical software version 20.0 (SPSS, Inc) and R statistical software version 4.1.2 (R Project for Statistical Computing). We used the weighted κ score^43^ to measure the degree of GWG classification agreement between the CNS 2021 and NAM 2009 guidelines, and prevalence rates were compared using χ^2^ tests. Two-sided *P* < .05 was considered significant. Risk ratio (RR) values were calculated by using modified Poisson regression models,^44^ and comparisons between the RRs were executed using heterogeneity tests. To control for potential bias, confounders were adjusted in the regression models. We ultimately calculated the sensitivity, specificity, positive predictive value (PPV), and negative predictive value (NPV) for appropriate GWG in estimating healthy nutritional status.

## Results

### Participants and Cohort Follow-up

A total of 3822 children (1996 boys and 1826 girls) with a mean (SD) age of 3.79 (0.30) years were recruited from the kindergarten junior class as our baseline population. They were enrolled and completed physical examinations that included body composition analysis between October 2017 and October 2018 at baseline. After excluding 74 twins or other multiple-births, 133 premature births, 29 postmature births, and 419 children who lacked maternal GWG data in the medical records of the antenatal health care system, we accepted 3170 term singleton children who possessed complete maternal GWG data in the antenatal health care system, and these provided the cohort baseline data. When the children were followed up through the kindergarten middle-class, 135 could not be contacted; thus, 3035 remained in the study. In the senior kindergarten class, 2274 children were followed up and had complete physical examination data (Figure).

**Figure.**
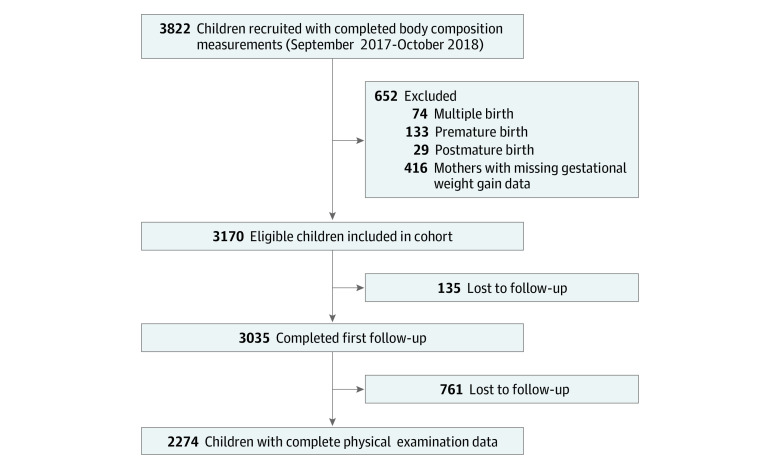
Flowchart of the Study Cohort

### Characteristics of the Participants

The proportion of women with a maternal pre-pregnancy BMI greater than or equal to 28 was 6.9% (220 of 3170 women), and the proportion with a prepregnancy BMI greater than or equal to 30 was 3.2% (100 of 3170 women). The mean (SD) values were 22.3 (3.4) for maternal prepregnancy BMI and 11.9 (4.1) kg for GWG. The mean (SD) values were 3412.1 (416.2) g for birth weight and 39.1 (1.1) weeks for gestational age. The prevalence rate for low birth weight was 0.8% (24 of 3170 children), and that for macrosomia was 9.2% (292 of 3170 children). For the children assessed during the 3-year follow-up in kindergarten, the mean values and ranges for BMI, FMI, FFMI, and FM% are shown in Table 1, and the prevalence rates regarding low and high nutritional statuses are also depicted.

**Table 1.** Characteristics of the Mothers and Their Preschool Offspring

Characteristics	Participants, No. (%) [95% CI]
Mothers	
Prepregnancy BMI, mean (SD) [range] (n = 3170)^a^	22.3 (3.4) [14.5 to 43.0]
Gestational weight gain, mean (SD) [range], kg (n = 3170)	11.9 (4.1) [−4.5 to 32.0]
Maternal age, mean (SD) [range], y (n = 3149)	28.9 (3.3) [18.0 to 43.0]
Parity	
1	2748 (86.7) [85.5 to 87.8]
2	410 (12.9) [11.8 to 14.1]
3	12 (0.4) [0.2 to 0.6]
Children	
Birth weight, mean (SD) [range], g (n = 3170)	3412.1 (416.2) [1945.0 to 5330.0]
Low birth weight	24 (0.8) [0.5 to 1.1]
Macrosomia	292 (9.2) [8.2 to 10.3]
Breast-fed for 6 mo	
Yes	1999 (63.6) [62.0 to 65.3]
No	1121 (35.7) [34.0 to 37.4]
Gestational age, mean (SD) [range], wk (n = 3170)	39.1 (1.1) [37.0 to 41.0]
BMI, mean (SD) [range]^a^	
3 y (n = 3170)	15.6 (1.4) [11.3 to 26.3]
4 y (n = 3009)	15.6 (1.6) [11.3 to 25.9]
5 y (n = 2274)	15.7 (2.0) [11.7 to 29.0]
FM%, mean (SD) [range]	
3 y (n = 3168)	18.0 (5.6) [3.0 to 48.0]
4 y (n = 3035)	16.8 (6.0) [3.0 to 44.7]
5 y (n = 2274)	16.6 (7.0) [3.0 to 47.8]
FMI, mean (SD) [range]	
3 y (n = 3168)	2.9 (1.2) [0.3 to 12.6]
4 y (n = 3009)	2.7 (1.3) [0.3 to 11.5]
5 y (n = 2274)	2.7 (1.6) [0.4 to 13.9]
FFMI, mean (SD) [range]	
3 y (n = 3168)	12.7 (0.7) [8.3 to 17.8]
4 y (n = 3009)	12.9 (0.7) [10.8 to 16.1]
5 y (n = 2274)	13.0 (0.8) [10.0 to 17.1]
3 y Low HAZ	216 (6.8) [6.0 to 7.7]
3 y Low WHZ/BAZ	362 (11.4) [10.4 to 12.6]
4 y Low HAZ	190 (6.3) [5.5 to 7.2]
4 y Low WHZ/BAZ	386 (13.4) [12.2 to 14.7]
5 y Low HAZ	88 (4.4) [3.5 to 5.3]
5 y Low BAZ	275 (13.6) [12.2 to 15.2]
3 y With obesity	35 (1.1) [0.8 to 1.5]
4 y With obesity	65 (2.2) [1.7 to 2.7]
5 y With obesity	146 (7.2) [6.2 to 8.4]
High AUC-FM%	422 (13.9) [12.7 to 15.1]
High AUC-FMI	373 (12.3) [11.1 to 13.5]
Low AUC-FFMI	458 (15.1) [13.8 to 16.4]

Abbreviations: AUC-FFMI, area under the curve of fat-free mass index in the 3 years’ follow-up; AUC-FM%, area under the curve of percentage of body fat in the 3 years’ follow-up; AUC-FMI, area under the curve of fat mass index in the 3 years’ follow-up; BAZ, *z* score of BMI for age; BMI, body mass index; HAZ, *z* score of height for age; WHZ, *z* score of weight for height.

^a^ BMI is calculated as weight in kilograms divided by height in meters squared.

### GWG Classification Under the CNS and NAM Guidelines

The GWG range and the recommended weekly weight gain values for singleton pregnancies under different prepregnancy BMIs are presented for CNS 2021 and NAM 2009 (eTable 1 in the Supplement); note that the recommendations in the NAM 2009 guidelines are numerically higher than those in the CNS 2021 guidelines. eTable 2 in the Supplement reveals that the prevalence rates were 14.1% (446 women) for insufficient GWG, 48.1% (1524 women) for appropriate GWG, and 37.9% (1200 women) for excessive GWG according to CNS 2021 guidelines, whereas the rates according to NAM 2009 guidelines were 39.7% (1259 women) for insufficient GWG, 37.2% (1180 women) for appropriate GWG, and 23.1% (731 women) for excessive GWG. A total of 1901 mothers (60.0%) were entered into the same GWG group using the 2 guidelines, and 1269 more mothers (40.0%) were placed into the higher GWG groups under the CNS guidelines than under the NAM guidelines. The weighted κ value of the GWG classification agreement between the CNS 2021 and NAM 2009 guidelines was 0.530 (95% CI, 0.510-0.550; *P* < .001).

### Children’s Health Outcomes Under the CNS and NAM Guidelines

The prevalence rates for low and high nutritional statuses, including birth weight and body composition evaluation in kindergarten, are depicted in Table 2. None of the prevalence rates for the children’s nutritional indices in the insufficient and excessive GWG groups differed between the 2 guidelines. For the appropriate GWG group, the rates of high nutritional levels were significantly lower under CNS guidelines than under NAM guidelines, but the rates for low nutritional levels were not different between the 2 guidelines.

**Table 2.** Children’s Health Outcomes Under the CNS and the NAM GWG Guidelines in Different Maternal GWG Groups

Children’s health outcomes	Insufficient maternal GWG			Appropriate maternal GWG			Excessive maternal GWG		
	Children, No. (%)		*P* value	Children, No. (%)		*P* value	Children, No. (%)		*P* value
	CNS (n = 446)	NAM (n = 1259)		CNS (n = 1524)	NAM (n = 1180)		CNS (n = 1200)	NAM (n = 731)	
Low nutrition status									
Low birth weight	5 (1.1)	13 (1.0)	.88	16 (1.0)	9 (0.8)	.44	3 (0.3)	2 (0.3)	.92
3 y Low HAZ	47 (10.5)	100 (7.9)	.09	110 (7.2)	77 (6.5)	.48	59 (4.9)	39 (5.3)	.68
3 y Low WHZ/BAZ	62 (13.9)	168 (13.3)	.77	194 (12.7)	140 (11.9)	.51	106 (8.8)	54 (7.4)	.26
4 y Low HAZ	41 (9.7)	88 (7.4)	.13	88 (6.1)	62 (5.6)	.58	61 (5.3)	40 (5.7)	.76
4 y Low WHZ/BAZ	73 (18.4)	182 (16.1)	.29	206 (14.9)	144 (13.4)	.30	107 (9.8)	60 (8.9)	.55
5 y Low HAZ	16 (5.5)	42 (5.2)	.89	47 (4.9)	30 (4.0)	.40	25 (3.3)	16 (3.4)	.90
5 y Low BAZ	53 (18.1)	130 (16.2)	.47	145 (15.0)	99 (13.2)	.29	77 (10.2)	46 (9.9)	.86
Low AUC-FFMI	81 (18.8)	216 (17.9)	.67	223 (15.3)	144 (12.8)	.07	154 (13.3)	98 (13.7)	.79
High nutrition status									
Macrosomia	19 (4.3)	61 (4.8)	.62	95 (6.2)	103 (8.7)	.01	178 (14.8)	128 (17.5)	.12
3 y With obesity	2 (0.4)	4 (0.3)	.69	7 (0.5)	15 (1.3)	.02	26 (2.2)	16 (2.2)	.97
4 y With obesity	6 (1.4)	11 (0.9)	.39	16 (1.1)	29 (2.6)	.004	43 (3.8)	25 (3.6)	.81
5 y With obesity	13 (4.4)	33 (4.1)	.81	55 (5.7)	61 (8.1)	.04	78 (10.3)	52 (11.2)	.64
High AUC-FM%	47 (10.9)	121 (10.0)	.60	168 (11.6)	167 (14.9)	.01	207 (17.9)	134 (18.8)	.62
High AUC-FMI	45 (10.5)	106 (8.8)	.31	143 (9.8)	144 (12.8)	.02	185 (16.0)	123 (17.2)	.48

Abbreviations: AUC-FFMI, area under the curve of fat-free mass index in the 3 years’ follow-up; AUC-FM%, area under the curve of percentage of body fat in the 3 years’ follow-up; AUC-FMI, area under the curve of fat mass index in the three years’ follow-up; BAZ, *z* score of body mass index for age; CNS, Chinese Nutrition Society; GWG, gestational weight gain; HAZ, *z* score of height for age; NAM, US National Academy of Medicine; WHZ, *z* score of weight for height.

### Diagnostic Ability of the CNS and the NAM Guidelines

eTable 3 in the Supplement shows the results of the associations between multiple offspring outcomes and GWG categories based on the CNS 2021 and the NAM 2009 GWG guidelines. We used modified Poisson regression models adjusted for prepregnancy BMI, maternal age at delivery, mother’s and father’s careers, mother’s and father’s educational levels, annual family income, parity, gestational age, and whether the mother had breast-fed for 6 months, and we then calculated the RRs (95% CIs) for the children of mothers with insufficient and excessive GWG and those who manifested a low or high nutritional level compared with the appropriate GWG group.

We observed that compared with pregnant women with adequate GWG as judged using CNS 2021 guidelines, insufficient GWG was positively associated with the risk for low height-for-age *z* score at age 4 years (RR, 1.49; 95 CI%, 1.02-2.17) and was not associated with other unhealthy nutritional outcomes of the offspring, whereas excessive GWG was positively associated with risk in all the high nutritional-level indices of the offspring, including macrosomia; obesity at age 3, 4, and 5 years; and high FM% and high FMI at 3 to 5 years, with RRs between 1.28 and 4.47.

The 95% CI ranges of the RRs calculated using the CNS 2021 and the NAM 2009 GWG guidelines for unhealthy nutritional status in offspring all overlapped. We then used heterogeneity tests to compare the RRs between the 2 guidelines, and none were significant, although the RRs in correlating obesity in 3-year-olds and 4-year-olds in the excessive GWG group appeared to be numerically different between the 2 guidelines.

The sensitivity, specificity, PPV, and NPV for appropriate GWG based on the CNS 2021 and NAM 2009 guidelines that corresponded to healthy nutritional status (including nonlow nutritional status and nonhigh nutritional status) are shown in Table 3. The CNS guidelines revealed higher sensitivity values for appropriate GWG in estimating the nonlow nutritional status of the offspring than did the NAM guidelines, whereas the specificity, PPV, and NPV values were all numerically higher according to the NAM guidelines compared with those of the CNS guidelines. On the basis of the 2 guidelines, the PPVs of appropriate GWG in estimating the nonlow nutritional status of offspring were all above 0.847 (range, 0.847-0.992), and the NPVs were all less than 0.164 (range, 0.005-0.164).

**Table 3.** Sensitivity, Specificity, PPV, and NPV for Appropriate Gestational Weight Gain Corresponding to Each Target Event, Under CNS 2021 and NAM 2009 Guidelines, Respectively

Target event and guidelines	Sensitivity (95% CI)	Specificity (95% CI)	PPV (95% CI)	NPV (95% CI)
Nonlow nutrition status				
Nonlow birth weight				
NAM	0.372 (0.355-0.389)	0.625 (0.426-0.796)	0.992 (0.986-0.996)	0.008 (0.004-0.014)
CNS	0.479 (0.462-0.497)	0.333 (0.172-0.532)	0.990 (0.983-0.994)	0.005 (0.002-0.009)
3 y Nonlow HAZ				
NAM	0.373 (0.356-0.391)	0.644 (0.578-0.705)	0.935 (0.920-0.948)	0.070 (0.059-0.082)
CNS	0.478 (0.461-0.497)	0.491 (0.425-0.557)	0.928 (0.914-0.940)	0.064 (0.053-0.077)
3 y Nonlow WHZ/BAZ				
NAM	0.370 (0.352-0.388)	0.613 (0.562-0.662)	0.881 (0.862-0.899)	0.112 (0.098-0.126)
CNS	0.474 (0.455-0.492)	0.464 (0.413-0.516)	0.873 (0.855-0.889)	0.102 (0.088-0.118)
4 y Nonlow HAZ				
NAM	0.372 (0.354-0.390)	0.674 (0.605-0.737)	0.944 (0.929-0.957)	0.067 (0.057-0.079)
CNS	0.480 (0.461-0.498)	0.537 (0.466-0.607)	0.939 (0.926-0.950)	0.065 (0.054-0.078)
4 y Nonlow WHZ/BAZ				
NAM	0.373 (0.354-0.392)	0.627 (0.578-0.674)	0.866 (0.844-0.885)	0.134 (0.119-0.150)
CNS	0.473 (0.452-0.492)	0.466 (0.417-0.516)	0.851 (0.832-0.869)	0.121 (0.105-0.138)
5 y Nonlow HAZ				
NAM	0.374 (0.352-0.396)	0.659 (0.556-0.752)	0.960 (0.944-0.972)	0.046 (0.035-0.058)
CNS	0.478 (0.456-0.500)	0.466 (0.364-0.570)	0.951 (0.937-0.964)	0.039 (0.029-0.052)
5 y Nonlow BAZ				
NAM	0.374 (0.352-0.397)	0.640 (0.582-0.695)	0.868 (0.843-0.891)	0.139 (0.121-0.159)
CNS	0.473 (0.450-0.496)	0.473 (0.414-0.532)	0.850 (0.827-0.872)	0.124 (0.105-0.145)
Nonlow AUC-FFMI				
NAM	0.379 (0.360-0.397)	0.686 (0.642-0.727)	0.872 (0.851-0.890)	0.164 (0.148-0.181)
CNS	0.476 (0.457-0.495)	0.513 (0.467-0.559)	0.847 (0.827-0.864)	0.148 (0.131-0.166)
Nonhigh nutrition status				
Nonmacrosomia				
NAM	0.374 (0.357-0.392)	0.647 (0.591-0.700)	0.913 (0.896-0.928)	0.095 (0.083-0.108)
CNS	0.497 (0.478-0.515)	0.675 (0.619-0.726)	0.938 (0.925-0.949)	0.120 (0.105-0.136)
3 y Without obesity				
NAM	0.371 (0.355-0.388)	0.571 (0.407-0.724)	0.987 (0.980-0.993)	0.001 (0.000-0.015)
CNS	0.484 (0.466-0.501)	0.800 (0.647-0.906)	0.995 (0.991-0.998)	0.017 (0.012-0.024)
4 y Without obesity				
NAM	0.367 (0.350-0.385)	0.554 (0.433-0.670)	0.974 (0.963-0.982)	0.019 (0.014-0.026)
CNS	0.484 (0.466-0.502)	0.754 (0.640-0.846)	0.989 (0.982-0.993)	0.031 (0.024-0.041)
5 y Without obesity				
NAM	0.369 (0.347-0.391)	0.582 (0.501-0.660)	0.919 (0.898-0.937)	0.067 (0.054-0.082)
CNS	0.489 (0.466-0.511)	0.623 (0.543-0.699)	0.943 (0.927-0.957)	0.087 (0.071-0.105)
Nonhigh AUC-FM%				
NAM	0.365 (0.347-0.383)	0.604 (0.557-0.650)	0.851 (0.829-0.871)	0.133 (0.118-0.149)
CNS	0.491 (0.472-0.510)	0.602 (0.555-0.648)	0.884 (0.867-0.900)	0.160 (0.143-0.179)
Nonhigh AUC-FMI				
NAM	0.367 (0.348-0.385)	0.614 (0.564-0.662)	0.872 (0.851-0.890)	0.119 (0.105-0.134)
CNS	0.491 (0.472-0.510)	0.617 (0.567-0.665)	0.902 (0.885-0.916)	0.145 (0.128-0.163)

Abbreviations: AUC-FFMI, area under the curve of fat-free mass index in the 3 years’ follow-up; AUC-FM%, area under the curve of percentage of body fat in the 3 years’ follow-up; AUC-FMI, area under the curve of fat mass index in the 3 years’ follow-up; BAZ, *z* score of body mass index for age; CNS, Chinese Nutrition Society; HAZ, *z* score of height for age; NAM, US National Academy of Medicine; NPV, negative predictive value; PPV, positive predictive value; WHZ, *z* score of weight for height.

When the sensitivity, specificity, PPV, and NPV of mothers who maintained appropriate GWG during pregnancy were used to estimate the nonhigh nutritional status of their offspring according to CNS guidelines, we noted that the values were numerically higher than those based on the NAM recommendations (except for the specificities of the 2 guidelines in the nonhigh AUC-FM% group, which were similar at 0.604 and 0.602). The PPVs with respect to appropriate GWG in estimating the nonhigh nutritional status of offspring were also all above 0.851 (range, 0.851-0.995), and the NPVs were all below 0.160 (range, 0.001-0.160) based on the 2 guidelines.

## Discussion

Asian individuals are typically of shorter stature than individuals from most Western countries, and there are different cutoffs for BMI categories in adults.^45^ In addition, Asian individuals generally possess a lower BMI but higher percentage of body fat than do White individuals,^46^ and tend to be more susceptible to metabolic complications even at lower BMIs.^23^ Chinese individuals differ considerably from Western individuals in terms of body size and metabolic levels, and the effects of GWG on the health of mothers and offspring may also vary by race. Considering the variations in race, ethnicity, dietary habits, and other factors between Chinese and Western populations, large representative test samples are required to assess optimal GWG recommendations as they pertain to adverse perinatal outcomes and that are relevant to local populations.^47^

Several studies in China have in recent years explored the GWG range suitable for Chinese women. Wang et al^48^ investigated the appropriate GWG range corresponding to Chinese-specific BMI categories and estimated its association with offspring birthweight according to the data of 16 460 healthy pregnant women in Beijing. Zhang et al^49^ sought to establish optimal GWG for Chinese pregnant women by applying Chinese-specific BMI categories through a multicenter, prospective cohort study that involved data from 3731 singleton pregnancies and compared these new recommendations with the NAM 2009 guidelines. With the establishment of a cohort that included more than 100 000 pregnant women from 9 provinces in China and using article summaries published by different Chinese authors that comprised a total sample size of more than 247 000 individuals, a new GWG group standard was formulated, evaluated, and promulgated as a group standard by the CNS in 2021.^36^ The CNS thus indicated that this standard could be widely used in the weight management of perinatal health care in China.

A meta-analysis^34^ in which the authors monitored GWG and prepregnancy BMI and that applied the 2009 NAM guidelines to the global population revealed that the lowest mean prepregnancy BMI was found in Asia at 21.24 (95% CI, 20.76-21.71) and that the lowest mean GWG was also in Asia at 11.36 kg (95% CI, 10.14-12.58 kg), results similar to those in our study (22.3 and 11.9 kg, respectively). Compared with the pooled prevalence rates in Asian population based on NAM 2009 guidelines,^34^ the total prevalence of insufficient GWG in our study was similar (39.7% vs 39.4%), and the rate of excessive GWG in our sample was higher (23.1% vs 16.8%).

Our cohort study showed that the maternal GWG classification agreement between the CNS 2021 and NAM 2009 guidelines was moderate (weighted κ, 0.530), with the GWG recommendations given by the NAM higher than those provided by the CNS. On the basis of the health outcomes of the offspring, our analysis indicated that the children of Chinese mothers whose GWG was appropriate as defined by CNS 2021 guidelines exhibited lower prevalence rates for macrosomia, obesity, high FM%, and high FMI between ages 3 and 5 years compared with those whose GWG was defined as appropriate under NAM guidelines, without simultaneously increasing the prevalence rates of low nutritional status. According to the 2 guidelines, the PPVs with respect to appropriate GWG in estimating nonlow nutritional status and nonhigh nutritional status of offspring were all above 0.84, and the NPVs were all less than 0.17. Compared with pregnant women who demonstrated adequate GWG as judged using the CNS 2021 guidelines, excessive GWG was positively associated with risk for all the high nutritional status indices of the offspring, with RRs between 1.28 and 4.47. Although optimal GWG recommendations may be used to inform prenatal counseling, the GWG guidelines exacted no effect on weight gain among pregnant women. We therefore recommend that policies targeting maternal and child health be established as routine antenatal care and policy worldwide.^50,51^

To our knowledge, this is the first report in which the applicability of the national GWG recommendations in China was assessed after the CNS guidelines were released. Furthermore, not only were the children’s health outcomes under the CNS and the NAM guidelines compared, but the diagnostic abilities of the 2 guidelines were also evaluated.

### Strengths and Limitations

The data used in this report possess their own strengths. For example, both the continuous follow-up of child adiposity development in the 3 years of kindergarten and data from obstetric records during pregnancy were included in a bidirectional cohort study. Moreover, maternal gestational weight was measured at antenatal clinics at each visit, and the medical records of the gestational weight improved the reliability of the GWG data.

There were also some limitations to the present study. First, only data from Tianjin were adopted in the comparison of the 2 guidelines, and additional multicenter studies with larger sample sizes are needed to verify the reliability of CNS recommendations among Chinese women. Second, we suggest that other specific adverse outcomes, such as preterm delivery, stillbirth, neonatal death, congenital malformation, gestational diabetes, and maternal postpartum weight loss, be considered in the evaluation of GWG guidelines. Third, prepregnancy weight was self-reported during the first visit to the antenatal clinic, and a recall bias of the self-reported weight data may have taken place. Although a previous systematic review concluded that self-reported pregnancy-related weight was highly correlated with weight measurements,^52^ women generally underestimated their own weights,^53^ and this may have led to a higher than actual GWG. Fourth, we measured the children’s adiposity indices using the BIA method rather than the reference-standard measure, thus improving feasibility, but its accuracy requires further consideration. There is strong evidence for the acceptable reliability of the BIA, but it is susceptible to considerable measurement error.^54^ In addition, lifestyle behaviors, such as maternal smoking, diet, sleep, and physical activity, might act as confounding factors in the association between pregnancy weight and offspring body composition. However, since these indices were not collected in an ideal fashion, they were not adjusted in the final analysis.

## Conclusions

In this study, it was found that the GWG recommendations provided by the NAM were higher than those of the CNS. Thus, monitoring the GWG of Chinese women within the appropriate range recommended by the CNS could facilitate a reduction in the risk of macrosomia and preschool obesity in their offspring. Accordingly, CNS guidelines for GWG may be more suitable than NAM guidelines for Chinese women.

## Notes

Supplement. **eTable 1.** The Chinese Nutrition Society (CNS 2021) and the American Institute of Medicine (NAM 2009) Guidelines: Recommendations for Total and Rate of Weight Gain During Pregnancy, by Prepregnancy BMI **eTable 2.** The Distribution of Mothers According to the CNS and NAM Gestational Weight Gain Guidelines, *n* (%) **eTable 3.** Relative Risk [RRs (95% CIs)] of the Children of Insufficient and Excessive GWG Mothers to Having a Low/High Nutrition Level, Compared With the Appropriate GWG Group
