# Supplementary material for: Analysis and Comparison of Early Childhood Nutritional Outcomes Among Offspring of Chinese Women Under the Chinese 2021 and US 2009 Gestational Weight Gain Guidelines
Source: JAMA Netw Open. 2022 Sep 23;5(9):e2233250. doi: 10.1001/jamanetworkopen.2022.33250 (PMC9508653; doi:10.1001/jamanetworkopen.2022.33250)
Supplement: Supplement. — eTable 1. The Chinese Nutrition Society (CNS 2021) and the American Institute of Medicine (NAM 2009) Guidelines: Recommendations for Total and Rate of Weight Gain During Pregnancy, by Prepregnancy BMI eTable 2. The Distribution of Mothers According to the CNS and NAM Gestational Weight Gain Guidelines, n (%) eTable 3. Relative Risk [RRs (95% CIs)] of the Children of Insufficient and Excessive GWG Mothers to Having a Low/High Nutrition Level, Compared With the Appropriate GWG Group [file jamanetwopen-e2233250-s001.pdf]

Supplemental Online Content

Chen F, Wang P, Wang J, et al. Analysis and comparison of early childhood nutritional outcomes among offspring of Chinese women under the Chinese 2021 and US 2009 gestational weight gain guidelines. *JAMA Netw Open.* 2022;5(9):e2233250. doi:10.1001/jamanetworkopen.2022.33250

eTable 1. The Chinese Nutrition Society (CNS 2021) and the American Institute of Medicine (NAM 2009) Guidelines: Recommendations for Total and Rate of Weight Gain During Pregnancy, by Prepregnancy BMI
eTable 2. The Distribution of Mothers According to the CNS and NAM Gestational Weight Gain Guidelines, *n* (*%*)
eTable 3. Relative Risk [RRs (95% CIs)] of the Children of Insufficient and Excessive GWG Mothers to Having a Low/High Nutrition Level, Compared With the Appropriate GWG Group

This supplemental material has been provided by the authors to give readers additional information about their work.

© 2022 Chen F et al. *JAMA Network Open.*

eTable 1. The Chinese Nutrition Society (CNS 2021) and the American Institute of Medicine (NAM 2009) Guidelines: Recommendations for Total and Rate of Weight Gain During Pregnancy, by Prepregnancy BMI			
Prepregnancy BMI	Total weight gain range (kg)	Rates of weight gain in the first trimester (kg)	Rates of weight gain in the 2nd and 3rd trimesters (kg/week)
CNS 2021			
Underweight ($BMI<18.5kg/m^2$)	11.0~16.0	0~2.0	0.46 (0.37~0.56)
Normal weight ($18.5kg/m^2 \le BMI<24.0kg/m^2$)	8.0~14.0	0~2.0	0.37 (0.26~0.48)
Overweight ($24.0kg/m^2 \le BMI<28.0kg/m^2$)	7.0~11.0	0~2.0	0.30 (0.22~0.37)
Obese ($BMI \ge 28.0kg/m^2$)	5.0~9.0	0~2.0	0.22 (0.15~0.30)
NAM 2009			
Underweight ($BMI<18.5kg/m^2$)	12.5~18.0	0.5~2.0	0.51 (0.44~0.58)
Normal weight ($18.5kg/m^2 \le BMI<25.0kg/m^2$)	11.5~16.0	0.5~2.0	0.42 (0.35~0.50)
Overweight ($25.0kg/m^2 \le BMI<30.0kg/m^2$)	7.0~11.5	0.5~2.0	0.28 (0.23~0.33)
Obese ($BMI \ge 30.0kg/m^2$)	5.0~9.0	0.5~2.0	0.22 (0.17~0.27)

© 2022 Chen F et al. *JAMA Network Open.*

eTable 2. The Distribution of Mothers According to the CNS and NAM Gestational Weight Gain Guidelines, *n* (%)

		NAM guidelines		
		Insufficient (1259, 39.7%)	Appropriate (1180, 37.2%)	Excessive (731, 23.1%)
CNS guidelines	Insufficient (446, 14.1%)	446（14.1）	0	0
	Appropriate (1524, 48.1%)	800（25.2）	724（22.8）	0
	Excessive (1200, 37.9%)	13（0.4）	456（14.4）	731（23.1）

Abbreviations: CNS: Chinese Nutrition Society; NAM: the US National Academy of Medicine

© 2022 Chen F et al. *JAMA Network Open.*

eTable 3. Relative Risk [RRs (95% CIs)] of the Children of Insufficient and Excessive GWG Mothers to Having a Low/High Nutrition Level, Compared With the Appropriate GWG Group							
	Insufficient		*P**		Excessive		*P**
	CNS guidelines (n=446)	NAM guidelines (n=1259)			CNS guidelines (n=1200)	NAM guidelines (n=731)	
Low nutrition status							
Low birth weight	1.43(0.43-4.70)	1.46(0.65-3.30)	0.974		0.24(0.06-0.90)	0.35(0.06-2.19)	0.731
3 yrs-low HAZ	1.40(0.99-1.99)	1.21(0.90-1.64)	0.531		0.71(0.51-0.99)	0.87(0.59-1.30)	0.442
3 yrs-low WHZ/BAZ	1.06(0.81-1.39)	1.02(0.83-1.26)	0.819		0.84(0.67-1.06)	0.72(0.53-0.98)	0.438
4 yrs-low HAZ	1.49(1.02-2.17)	1.30(0.94-1.80)	0.600		0.87(0.62-1.24)	0.99(0.65-1.51)	0.663
4 yrs-low WHZ/BAZ	1.23(0.96-1.58)	1.11(0.91-1.36)	0.528		0.82(0.65-1.02)	0.83(0.62-1.11)	0.928
5 yrs-low HAZ	0.90(0.49-1.65)	1.32(0.84-2.09)	0.318		0.57(0.34-0.97)	0.67(0.34-1.31)	0.723
5 yrs-low BAZ	1.16(0.85-1.56)	1.16(0.91-1.50)	0.968		0.87(0.67-1.13)	1.01(0.72-1.40)	0.501
Low AUC-FFMI	1.22(0.96-1.55)	1.29(1.06-1.57)	0.732		0.99(0.82-1.20)	1.24(0.98-1.57)	0.732
High nutrition status							
Macrosomia	0.69(0.42-1.12)	0.64(0.47-0.87)	0.804		1.98(1.54-2.54)	1.78(1.38-2.31)	0.575
3 yrs-obese	0.85(0.21-3.43)	0.34(0.10-1.08)	0.314		4.47(1.92-10.42)	1.69(0.75-3.78)	0.103
4 yrs-obese	1.06(0.39-2.89)	0.46(0.22-0.97)	0.196		3.05(1.76-5.30)	1.37(0.76-2.45)	0.050

© 2022 Chen F et al. *JAMA Network Open.*

5 yrs-obese	0.72(0.40-1.30)	0.60(0.38-0.92)	0.607		1.45(1.00-2.08)	1.18(0.80-1.74)	0.455
High AUC-FM%	0.92(0.68-1.25)	0.81(0.65-1.01)	0.504		1.28(1.05-1.56)	1.12(0.90-1.39)	0.364
High AUC-FMI	1.03(0.74-1.42)	0.82(0.64-1.04)	0.273		1.33(1.07-1.65)	1.15(0.91-1.46)	0.381

Abbreviations: CNS: Chinese Nutrition Society; NAM: the US National Academy of Medicine; HAZ: z-score of height-for-age; WHZ: z-score of weight for height; BAZ: z-score of BMI-for-age; AUC-FFMI: area under the curve of fat free mass index in the three years' follow-up; AUC-FM%: area under the curve of percentage of body fat in the three years' follow-up; AUC-FMI: area under the curve of fat mass index in the three years' follow-up.

© 2022 Chen F et al. *JAMA Network Open*.
